# Impact of Suberin Depolymerization Conditions on the Thermal Properties and Flammability of Rigid Polyurethane Foams

**DOI:** 10.3390/polym18111355

**Published:** 2026-05-29

**Authors:** Aiga Ivdre, Mikelis Kirpluks, Daniela Godina, Arnis Abolins, Laima Vevere, Rudolfs Berzins, Maris Lauberts, Janis Rizikovs

**Affiliations:** Latvian State Institute of Wood Chemistry, LV-1006 Riga, Latvia; aiga.ivdre@kki.lv (A.I.); daniela.godina@kki.lv (D.G.); arnis.abolins@kki.lv (A.A.); laima.vevere@kki.lv (L.V.); rudolfs.berzins@kki.lv (R.B.); maris.lauberts@kki.lv (M.L.); janis.rizikovs@kki.lv (J.R.)

**Keywords:** suberin, suberinic acids, polyols, rigid polyurethane foams, flammability

## Abstract

Suberinic acids (SA) derived from birch outer bark are renewable feedstocks for bio-based polyols suitable for rigid polyurethane (PU) foams. Three SA fractions were prepared under different depolymerization conditions: acidification at pH 1 (SA1), pH 5 (SA2), and FeCl_3_-assisted treatment (SA3), and their chemical composition was analysed by GC–MS, Py–GC/MS, and GPC–RID. Polyols derived from tall oil fatty acids (TOFA) or epoxidized TOFA with trimethylolpropane were used as the sole polyol components in foam formulations. The SA fractions differed in molecular weight distribution, affecting polyol processability. All foams exhibited similar limiting oxygen index (19–20) and cone calorimetry results, showing no statistically significant differences in flammability. This indicates that variations in depolymerization conditions, including polyphenolic content and removal of higher-molecular-weight fractions during FeCl_3_ treatment, do not dominate fire performance under the studied conditions. SA3-based polyols showed the lowest viscosity and produced foams with optimal mechanical and thermal properties, while SA1 offered higher yield with comparable performance. These results demonstrate the feasibility of converting SA fractions into functional polyols for rigid PU foams and highlight the FeCl_3_-treated SA3 fraction and SA1 as the most promising candidates for further development.

## 1. Introduction

The growing demand for sustainable materials and the need to reduce reliance on fossil-based resources have intensified research into renewable feedstocks for polymer production [1]. Birch outer bark, an abundant by-product of the plywood and veneer industries, is particularly rich in suberin—a complex, cross-linked biopolyester composed primarily of long-chain aliphatic acids, alcohols, and phenolic components [2,3,4]. Despite its abundance, commercial utilization of bark-derived products remains limited [5]. Nevertheless, suberin and its depolymerization products—suberinic acids (SA)—have attracted increasing attention as renewable precursors for binders [6,7], adhesives [8], surfactants [9,10], and bio-based polyols for polyurethane (PU) foam production [11,12,13].

Rigid PU foams are widely employed in thermal insulation, construction, refrigeration, and packaging due to their low thermal conductivity, high compressive strength, and lightweight structure [14]. However, conventional PU foams rely predominantly on petroleum-derived polyols, raising environmental and sustainability concerns. Substituting fossil-based components with renewable alternatives—such as polyols derived from SA—is aligned with global initiatives to reduce carbon footprint and promote a circular bioeconomy [15,16].

SA, obtained via alkaline depolymerization of birch bark, form a chemically diverse mixture of aliphatic and aromatic compounds bearing multiple functional groups (–COOH, –OH, and –O–) [17]. These functionalities make SA suitable for polyol synthesis through esterification or transesterification reactions. Their composition, reactivity, and viscosity, however, depend strongly on depolymerization conditions (pH, temperature, catalyst type), which affect monomeric and oligomeric distribution, polyphenolic content, and overall molecular weight [18]. Polyphenols are particularly relevant, as they can enhance the fire retardancy of PU foams by promoting thermally stable char formation and acting as radical scavengers. For instance, tannic acid modified with iron ions (Fe^3+^) has been shown to improve PU flame resistance by facilitating char formation and reducing heat release rates [19]. Similarly, aromatic-rich polyester polyols can enhance compressive strength and thermal stability in rigid PU foams [20,21,22]. To reduce SA polyol viscosity while maintaining a high renewable content, tall oil fatty acids (TOFA)—derived from the byproduct of pulp mills—can be incorporated as a flexible aliphatic component, improving processability and foam performance [12,23].

Previous studies have shown that SA-based polyols, with or without added TOFA in polyol synthesis, can partially or fully substitute conventional petrochemical polyols in rigid PU foam formulations [11,12,18]. However, a comprehensive understanding of how depolymerization parameters influence the chemical structure of SA, the resulting polyol properties, and the PU foam performance remains limited—particularly regarding polyphenolic content, thermal stability, and flammability.

In this study, the influence of suberin depolymerization conditions on SA-derived polyols and rigid PU foams was evaluated, focusing on viscosity, mechanical strength, thermal stability, and flammability. Three SA fractions were prepared under distinct depolymerization regimes—acid precipitation at pH 1 and 5, and FeCl_3_-assisted treatment. Polyols were synthesized from these SA fractions together with TOFA, and rigid PU foams were subsequently fabricated and thoroughly characterized to assess the impact of SA depolymerization conditions and polyol composition on foam properties.

## 2. Materials and Methods

### 2.1. Experimental Design

**Figure 1:**
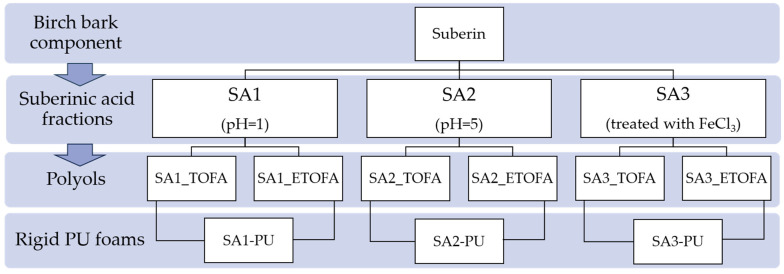
Process flow for suberin depolymerization, polyol synthesis, and rigid PU foam production.

The experimental design of this study comprises several key stages: suberin depolymerization and characterization; synthesis of polyols from SA and TOFA or epoxidized TOFA (ETOFA); characterization of the synthesized polyols; and the production and evaluation of rigid PU foams. To enhance clarity regarding the research workflow and sample designations, an overview is presented in Figure 1.

### 2.2. Suberin Depolymerization and Characterization of Suberinic Acids

SA were obtained from extracted birch outer bark [24] using alkaline depolymerisation in ethanol/water medium, followed by acidification and purification. The general workflow of bark preparation, depolymerisation in KOH/ethanol solution, and subsequent acidification has been described previously [11,17].

Potassium hydroxide (KOH) (Reag. Ph Eur, 85.0%) was provided by VWR International (Leuven, Belgium). Nitric acid (HNO_3_) (65%) was obtained from Honeywell (Seelze, Germany). Iron (III) chloride (FeCl_3_, Reagent grade, 97%) provided by Sigma-Aldrich (Darmstadt, Germany).

In this study, the procedure was scaled to 2500 g of extracted birch outer bark (1 ≤ d < 2 mm), depolymerised in a 30 L stainless steel reactor (original construction) equipped with a steam heating jacket, reflux condenser, and mechanical stirrer. The bark was suspended in a 4.3 wt% KOH ethanol solution at a bark-to-liquid mass ratio of 1:8, depolymerised at 78 °C for 30 min, then cooled to 30 °C and filtered through 100 μm non-woven polyamide fabric. SA salts were precipitated after removal of 70 wt% ethanol (Hei-VAP rotary evaporator, Heidolph Instruments GmbH & Co., Schwabach, Germany) and dilution with deionized water, followed by acidification with HNO_3_.

Three different SA fractions were prepared by varying the precipitation conditions:

1. SA1: obtained by adjusting the pH to 1 during acidification.

2. SA2: obtained by adjusting the pH to 5 during acidification.

3. SA3: prepared by treating the SA–salt ethanol filtrate obtained in the same procedure as for SA1. The only difference was that the cooled salt solution filtrate was treated with FeCl_3_ (18 g/L) under ambient atmosphere, without additional heating or the use of an inert gas. After the addition of FeCl_3_, the mixture pH was adjusted to 10 using KOH to precipitate phenolic compounds and higher-molecular-weight oligomers. The resulting FeCl_3_ complex solids were separated by vacuum filtration. The filtrate was then evaporated to 70 wt% of ethanol, diluted with deionized water, and acidified to pH 1 with HNO_3_ to precipitate SA.

All three SA suspensions were filtered under vacuum (4 L Büchner funnel, 10 L Bunsen flask, Grade 3 filter paper) to remove KNO_3_ salts and water-soluble impurities, washed with deionized water, and dried at 80 °C.

Analytical characterisation of SA fractions was carried out using established methods: GC–MS for monomeric composition (via direct silylation and depolymerisation before derivatisation) and GPC with RID/MALS detection for molar mass distribution, as reported by Godina et al. [17]. FTIR spectroscopy was used for functional group analysis, following the method of Godina et al. [17,24], and Py–GC/MS for determining total polyphenolic content as described by Lauberts et al. [25]. The acid value (H_v_) was determined according to ISO 2114:2000 [26].

### 2.3. Synthesis and Characterization of Polyols

SA-based polyols were synthesized from TOFA, SA fractions, and potassium hydroxide (KOH) as described by Ivdre et al. [12]. In total, six polyols were synthesized: SA1_TOFA, SA2_TOFA, SA3_TOFA, SA1_ETOFA, SA2_ETOFA, and SA3_ETOFA.

H_v_ and hydroxyl values (OH_v_) of the synthesized SA-based polyols were assessed in accordance with ISO 2114:2000 and ISO 4629-2:2016 standards, respectively [26,27]. The water content was determined by Karl Fischer titration according to ISO 760:1978 [11,28]. Apparent viscosity was measured at 25 °C using a cone–plate rheometer at a shear rate of 50 s^−1^, following the procedure described by Ivdre et al. [11].

SA-based polyols were characterized using the GPC-RID system as described in Section 2.2.

### 2.4. Rigid PU Foam Formulations

In addition to the synthesized polyols, several commercially available components were incorporated into the foam formulations. Catalysts PC CAT TKA 30^®^ and PC CAT NP10^®^ (Air Products and Chemicals Inc., Allentown, PA, USA) were used, while the surfactant Niax Silicone L-6915 was obtained from Momentive Performance Materials Inc. (Leverkusen, Germany). Opteon™ 1100 (Chemours, Wilmington, DE, USA) served as the physical blowing agent, and the distilled water was employed as the chemical blowing agent. The isocyanate component was Desmodur 44 V20 L (pMDI), a solvent-free 4,4′-diphenylmethane diisocyanate-based product containing high-functionality oligomers, supplied by Covestro (Augusta, GA, USA). The isocyanate exhibited an NCO content of 31.5 wt% and an average functionality of 2.8–2.9.

Rigid PU foam formulations, including polyol ratios, were selected based on the previous optimization reported by Ivdre et al. [12] with one exception; no flame retardant was added, allowing clearer evaluation of the polyols’ impact on the flammability of the rigid PU foams. The pMDI content was adjusted to maintain an NCO/OH ratio of 1.2. The formulations of rigid PU foams are shown in Table 1.

### 2.5. Rigid PU Foam Preparation and Characterization

Rigid PU foams were prepared as described previously by Ivdre et al. [12]. The polyol components were thoroughly mixed for approximately 2 min at a rate of 2000 rpm, followed by sequential mixing with Opteon™ 1100 for approximately 1 min at 2000 rpm and with pMDI for 10 s. The resulting reactive mixture was allowed to rise freely in the same container under ambient conditions. Foaming parameters (start time, gel time, and rise time) were recorded, and shrinkage was measured after 24 h. For larger-scale samples, the reactive mixture was poured into a 30 × 30 × 10 cm mould and allowed to cure at room temperature without external heating for 24 h.

The cellular structure of the PU foams was investigated using a light microscope Diamond MCXMP500 (MICROS Produktions- & Handels GmbH, Sankt Veit an der Glan, Austria) at a magnification of 5×.

The physical, thermal, mechanical, and flammability properties of the rigid PU foams were characterized following standard methods and established procedures described in previous studies. Apparent density and closed-cell content were measured according to ISO 845:2006 [29] and ISO 4590:2016 [30], respectively. Thermal conductivity (λ) was determined at an average temperature of 10 °C following ISO 8301:1991 [11,31]. Compressive strength and modulus were assessed on cylindrical specimens (≈20 mm diameter/height) using a Zwick/Roell Z100 (Zwick Roel, Ulm, Germany) universal testing machine according to ISO 844:2021 [32]. A 1 kN load cell was employed, and the deformation rate was set to 10%/min. The compressive strength was calculated either at the maximum stress or at 10% deformation, depending on the failure mode of the specimen. Tests were performed both parallel and perpendicular to the foam rise direction. The results were normalized to 40 kg/m^3^ as described by Hawkins et al. [33].

Dynamic mechanical analysis (DMA) was performed on a Mettler Toledo instrument (Mettler Toledo, Tokyo, Japan) to determine the glass transition temperature (T_g_), following testing regimes described by Ivdre et al. [12]. Thermal stability was evaluated by thermogravimetric analysis (TGA) using the Discovery TGA system (TA Instruments, New Castle, DE, USA) under nitrogen from 30 to 700 °C at 10 °C/min. All analyses were carried out in triplicate or more, and data were processed with TRIOS (v5.0.0.44608) and OriginPro 2021 (v9.8.0.200).

Flammability was assessed using a vertical small-flame and cone calorimeter tests according to ISO 11925-2:2020 and ISO 5660, respectively [34,35]. No specific control of temperature or relative humidity was applied during the tests. Rigid PU foam samples (3 parallel specimens of size 100 × 100 × 25 mm) were tested with a cone calorimeter (Fire Testing Technology, East Grinstead, UK) in a horizontal orientation at a 25 mm distance from the cone heater, under an applied heat flux of 35 kW/m^2^. Samples were placed in a stainless-steel retainer frame and wrapped with aluminium foil at the bottom and edges to ensure stability during testing. The following flammability parameters were recorded: time to ignition (TTI), time to flameout (TTF), total heat release (THR), peak heat release rate (pHRR), maximum average rate of heat emission (MAHRE), and total smoke release (TSR). The limiting oxygen index (LOI) was determined following the ISO 4589-2 standard [36]. Finally, the content of SA-based components and total renewable materials (SA, water, and TOFA) in the rigid PU foams was calculated as a weight fraction of the total foam mass.

## 3. Results and Discussion

### 3.1. Characterization of Suberinic Acid Fractions

**Figure 2:**
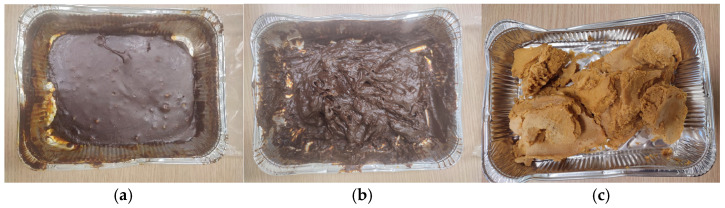
Visual appearance of SA: (**a**) SA1; (**b**) SA2; (**c**) SA3.

Three SA fractions were obtained under different conditions: SA1 (precipitated at pH1), SA2 (precipitated at pH5), and SA3 (treated with FeCl_3_ and precipitated at pH1). The visual appearance of the fractions is shown in Figure 2. Distinct differences were observed between the samples. SA1 and SA2 appeared as dark brown, dense materials, whereas SA3 exhibited a lighter, yellow-brown colour and a looser, more friable structure.

To characterize the SA samples, the dry mass content, yield, H_v_, and total polyphenolic compound content were determined using Py-GC/MS analysis, and the results are summarized in Table 2.

It was hypothesized that SA fractions with higher polyphenolic content could improve the flammability resistance and/or thermal properties of PU derived from SA-based polyols. Among the samples, SA1 exhibited the highest total polyphenolic content (3.84%), whereas SA3 showed the lowest (1.12%). In the case of SA3, treatment with FeCl_3_ resulted in the removal of both polyphenolic compounds and oligomeric fractions of SA. This effect is evident from the GC-MS and GPC analyses (Table 3 and Table 4), which reveal an increase in low-molecular-weight fractions as well as individual SA monomers. Consequently, the overall yield of SA3 was nearly two-fold lower than that of SA1. The higher acid number observed for SA3 after FeCl_3_ treatment may result from the removal of higher-molecular-weight oligomeric fractions, leading to an increased proportion of lower-molecular-weight species with a higher concentration of free carboxylic groups per unit mass. However, possible chemical modifications (e.g., hydrolysis or oxidation) could also be considered. 

**Figure 3:**
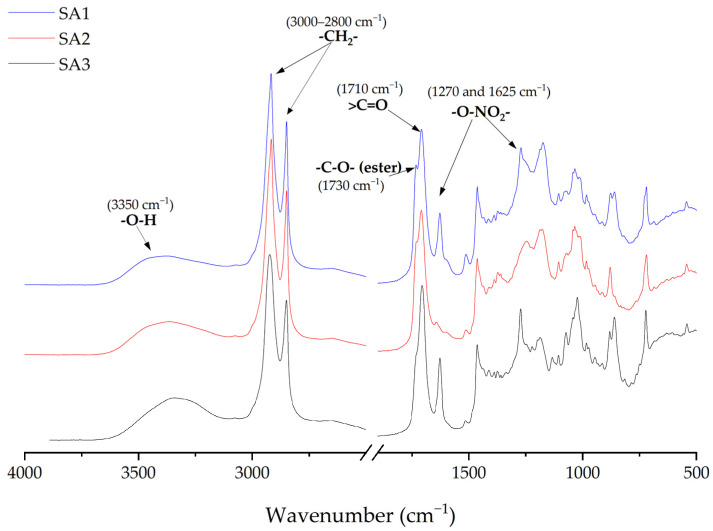
FTIR spectra of the SA fractions, showing characteristic functional groups.

To determine the key structural groups that indicate the suitability of SA fractions as polyol precursors for rigid PU foams, FTIR analysis was performed. The spectra obtained are presented in Figure 3.

Across all samples, a broad band near 3350 cm^−1^ corresponds to O–H stretching vibrations, indicating the presence of hydroxyl and carboxylic groups, while sharp peaks at 3000 cm^−1^ and 2800 cm^−1^ arise from aliphatic C–H stretching. Strong absorption at ~1730 cm^−1^ is attributed to ester carbonyls, whereas at 1710 cm^−1^ it reflects carboxylic acid carbonyls, both of which are essential for chemical reactivity during polyol synthesis. Aromatic C=C stretching bands (1600–1510 cm^−1^) confirm the presence of phenolic moieties [37,38], with a more pronounced peak in SA1, consistent with its higher phenolic content determined with Py-GC/MS/FID analysis. SA1 and SA3 also show residues of HNO_3_, evidenced by symmetric and asymmetric –O–NO_2_ stretching at 1270 cm^−1^ and 1625 cm^−1^, characteristic of SA fractions obtained at low pH [39]. The FTIR spectrum of SA3 is distinguished by stronger absorption in the carboxylic-type carbonyl region (~1710 cm^−1^) and a broader O–H band near 3350 cm^−1^ compared with SA1 and SA2. This pattern indicates a relative enrichment in free –COOH groups rather than absolute dominance, as SA1 and SA2 exhibit more pronounced ester shoulders around 1730 cm^−1^. These spectral differences align with the compositional data (GC/MS and GPC), which show that SA3 is enriched in acid-bearing fragments, whereas SA1 and SA2 contain more esterified oligomeric material. Consequently, SA3 represents the most reactive precursor for polyol synthesis, as its abundant carboxylic functionalities can be effectively converted into hydroxyl-bearing polyols.

**Figure 4:**
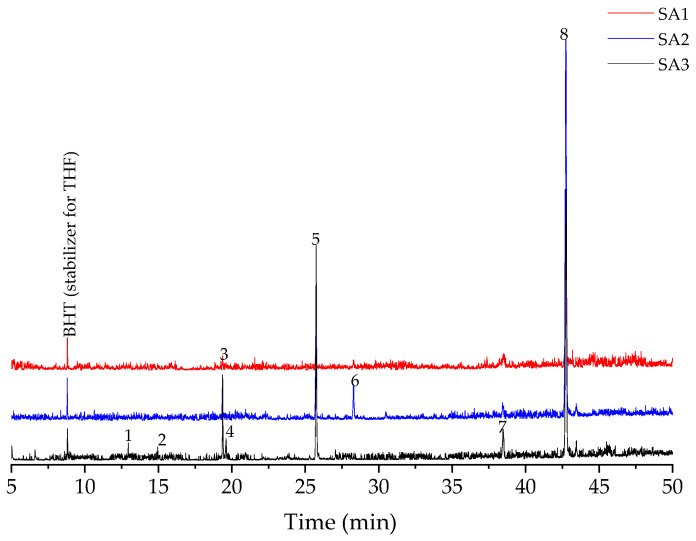
Total ion chromatogram of the SA samples. The peak number refers to the compounds listed in Table 3.

Suberin monomeric compounds were analysed by GC–MS. Quantification of individual monomers was performed based on the peak areas of the MS signals (Figure 4). GC–MS analysis of the trimethylsilylated derivatives demonstrated that the SA mixtures remain chemically diverse, yet exhibit clear compositional differences depending on the depolymerization conditions (Figure 4 and Table 3).

The main groups of compounds identified in the SA fractions were as follows:

1. Fatty acids and their esters—predominantly hexadecanoic acid methyl ester (C16:0) and ethyl stearate (C18:0), with minor amounts of eicosenoic (C20:1) and eicosadienoic acids (C20:2) detected in some samples, indicating partial cleavage of the aliphatic polyester backbone;
2. Epoxy and hydroxylated fatty acids—for example, octadecanoic acid, 9,10-epoxy-18-methyl ester observed in SA3, representing more oxidized aliphatic components;
3. Triterpenoid extractives—betulin was the dominant compound in all samples (78–82% in SA1–SA2, 54% in SA3), whereas lupeol was only detected in SA3, indicating selective enrichment depending on the extraction pathway.

Quantitative analysis revealed that the triterpenoid fraction dominated the composition of all samples, with betulin reaching up to 82% in SA1. The proportion of aliphatic esters and free fatty acids was highest in SA3, reflecting a shift towards more extensively depolymerized suberinic structures under its preparation conditions. The absence of detectable ω-hydroxyacids, diacids, and aromatic acids suggests either their removal during processing (e.g., drying) or concentrations below the detection limit for this analytical run. Despite these omissions, the mixture still contained both reactive aliphatic components and extractive-derived triterpenoids, highlighting that SA are multifunctional systems in which the balance between fatty acid derivatives and triterpenes is strongly influenced by the depolymerization protocol.

**Figure 5:**
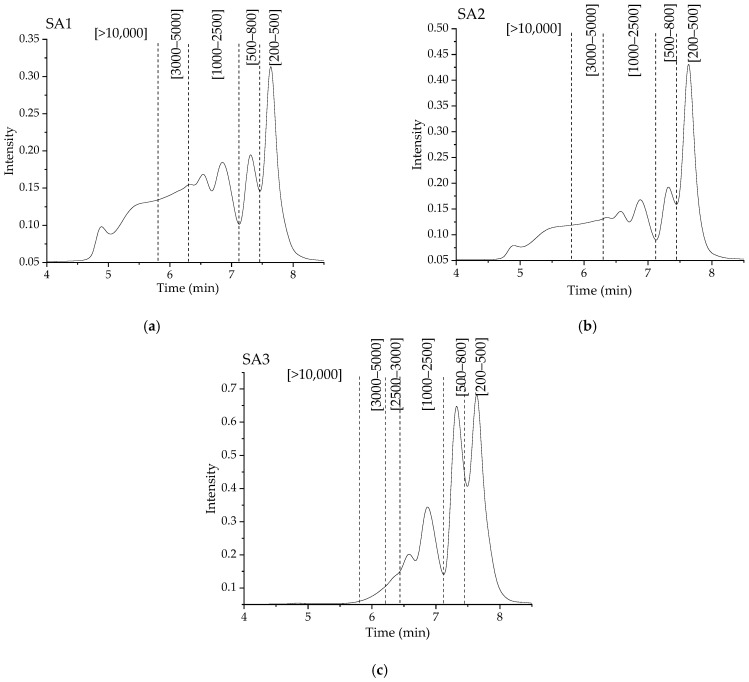
GPC-RID chromatograms of SA samples: (**a**) SA1; (**b**) SA2; (**c**) SA3.

Gel permeation chromatography with a refractive index detector (GPC-RID) provided detailed insight into the molecular weight distribution of the SA fractions. The chromatograms revealed a broad, polydisperse profile, indicating the presence of both low-molar-mass monomers/oligomers and a substantial proportion of higher-molar-mass oligomeric species (Figure 5).

For each analysed sample, deconvolution into distinct molecular weight populations was performed using the multiple peak fit function in OriginPro 2021b. The peak separation (fitting) procedure was based on the relationship between retention time and molecular weight obtained from the calibration with polystyrene standards. Polystyrene standards with molar masses of 200, 500, 850, 1000, 2500, 3000, 5000, 9000, 17,500, 20,000, and 30,000 g·mol^−1^ were used for calibration.

Because of structural differences between suberin-derived compounds and polystyrene standards, the obtained molecular weight values are only approximate. Therefore, the results are reported as molar mass ranges rather than absolute molar masses, since assigning exact molar masses based on polystyrene calibration would not be accurate.

The relative area (%) of each molecular weight population was calculated from the chromatographic peak areas using the calibration curve fitted with a third-order polynomial equation. The calculated values, based on polystyrene calibration, indicate that the SA fractions comprise a continuum of species ranging from <500 Da (monomeric acids and esters) to >10,000 Da, reflecting partially depolymerized oligomeric fragments (Table 4).

The low-molecular-weight peaks (200–500 Da) observed in GPC correspond to the monomeric species identified by GC–MS (e.g., fatty acids, diacids, and hydroxy acids). In addition, GPC reveals an oligomeric fraction (>500 Da) that cannot be resolved by GC–MS, confirming that a substantial portion of the SA is present as oligomeric polyesters. SA1 and SA2 exhibit similar molecular weight distributions, with more than one-third composed of oligomeric polyesters above 2500 Da, whereas SA3 predominantly contains those in the 200–2500 Da range. The monomeric and short oligomeric fractions provide readily available functional groups (–OH, –COOH) for PU chemistry, ensuring reactivity and crosslinking potential. The broad molecular weight distribution highlights the multifunctional nature of SA, bridging the roles of small reactive monomers and larger oligomeric structures in foam formation. Overall, GPC confirms that SA are not a uniform product but rather polydisperse mixtures of monomers and oligomers, whose combined presence is expected to influence the thermal behaviour, reactivity, and flammability of the resulting PU foams.

### 3.2. Characterization of SA-Based Polyols

#### 3.2.1. Physicochemical Properties

Two SA-based polyols were synthesized from each SA fraction—one using TOFA and the other using ETOFA—yielding a total of six polyols. Their key physicochemical properties are summarized in Table 5. Polyol stability was assessed by visual inspection. All polyols remained stable except for SA3_ETOFA, which showed signs of instability, evidenced by visible phase separation.

The SA content and the total share of renewable materials content reached approximately 35–37% and 71–75%, respectively. All polyols could be rendered fully bio-based if bio-based TMP were used in place of petrochemical TMP. Although commercially available TMP is still predominantly petrochemical, recent developments have demonstrated the feasibility of producing bio-based TMP from renewable feedstocks [40], and the market for such products is expected to expand [41]. These results underscore that the full bio-based potential of SA-derived polyols can be achieved when combined with renewable TMP.

Polyols synthesized with ETOFA exhibited higher OH_v_ (341–406 mg KOH/g) than those synthesized from unmodified TOFA (248–291 mg KOH/g), reflecting the greater reactivity of the epoxide groups in ETOFA, which introduce additional hydroxyl functionalities during the ring-opening reactions. All polyols had OH_v_ values within the typical range for rigid PU foam polyols (200–600 mg KOH/g), supporting the desired mechanical strength and thermal properties of the resulting foams [42,43]. Water content was low (≤0.04%) and accounted for in the foam formulations. Overall, the H_v_, OH_v_, and water content were within suitable ranges for rigid PU foam production.

Viscosity is a key factor influencing the practical use of polyols in both laboratory and industrial PU formulations. Polyols must have moderate viscosity to allow proper mixing, easy handling, and uniform cell structure formation during foaming. For rigid PU foams, polyol viscosities typically range from 500 to 5000 mPa·s at 25 °C. Excessively high viscosity (>10,000 mPa·s) can hinder processing, reduce flowability, and compromise foam quality, whereas too low viscosity (<300 mPa·s) may affect structural stability [22]. SA-based polyols have previously been reported to exhibit viscosities between 5 × 10^5^ and 7 × 10^7^ mPa·s [11,18], while incorporation of TOFA reduces viscosity to 120–2690 mPa·s [12]. Although polyols synthesized with ETOFA tend to have high viscosity, blending them with TOFA-based polyols yields a system with suitable processing characteristics. Among the samples studied, SA3-based polyols exhibited the lowest viscosity, reflecting the favourable effect of FeCl_3_ treatment on SA and indicating the best processing properties.

#### 3.2.2. Molar Mass Distribution in SA-Based Polyols

**Figure 6:**
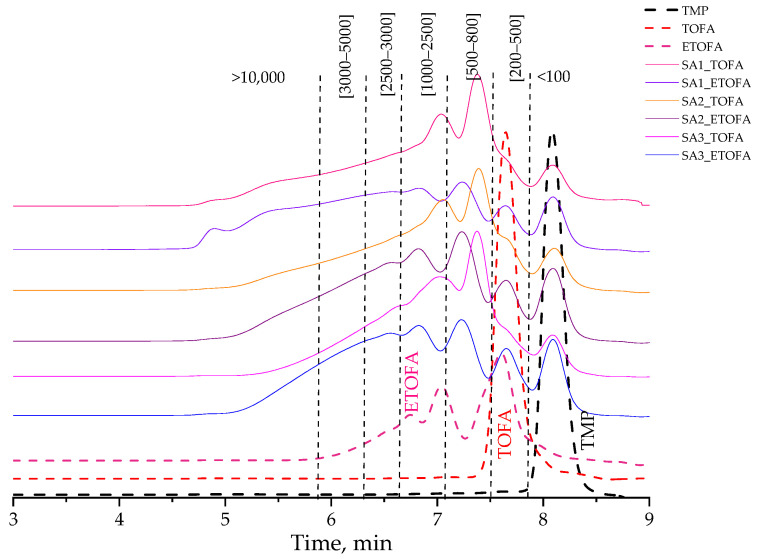
GPC-RID chromatograms of SA-based polyol samples.

The chemical composition of SA-based polyols was analysed by the GPC-RID system. The chromatograms (Figure 6) revealed clear differences depending on both the SA fraction and the type of fatty acid employed, which was either TOFA or ETOFA. The corresponding molar mass ranges and relative area percentages for the SA-based polyol samples are summarized in Table 6.

Polyols synthesized from ETOFA consistently exhibited higher-molecular-weight peaks than those derived from unmodified TOFA, indicating that epoxidation promotes chain extension and partial oligomerization during synthesis. The elevated hydroxyl values measured for ETOFA-based polyols do not directly correspond to higher molecular weights but instead reflect the introduction of additional hydroxyl functionalities through epoxide ring opening. As a result, ETOFA-based polyols possess both a greater number of reactive sites and a higher proportion of oligomeric species, which together contribute to their increased viscosity and enhanced crosslinking potential in PU formulation.

The origin of the SA fraction (SA1, SA2, SA3) strongly influenced the molecular weight distribution of the resulting polyols. SA1- and SA2-based polyols exhibited relatively broad distributions, reflecting the incorporation of both short-chain oligomers and longer, branched species. SA2-based polyols, in particular, showed slightly broader peak shapes, consistent with their intermediate phenolic content and heterogeneous reactivity. In contrast, SA3-based polyols—especially those synthesized with ETOFA—displayed a distinct high-molecular-weight shoulder indicative of more condensed oligomeric structures. Notably, phase separation was observed in the SA2_ETOFA system, likely due to its intermediate polarity and the imbalance between hydrophilic and hydrophobic components, which limited compatibility in polyol mixtures.

In all cases, a low-molecular-weight peak near the position of TMP was detected, indicating either residual initiator or short-chain oligomers. This peak was more pronounced in TOFA-based polyols, suggesting less efficient chain extension compared with ETOFA-based counterparts. Overall, GPC analysis confirms that SA-based polyols are highly polydisperse mixtures, with both the synthesis route (TOFA vs. ETOFA) and the origin of the SA fraction exerting a strong influence on the molecular weight distribution.

### 3.3. Characterization of SA-Based Rigid PU Foams

Rigid PU foams were prepared exclusively from the synthesized SA-based polyols, following the formulations summarized in Table 1 (Section 2). No additional flame retardants were added, allowing the SA-based polyols’ specific contribution to flammability to be assessed.

**Figure 7:**
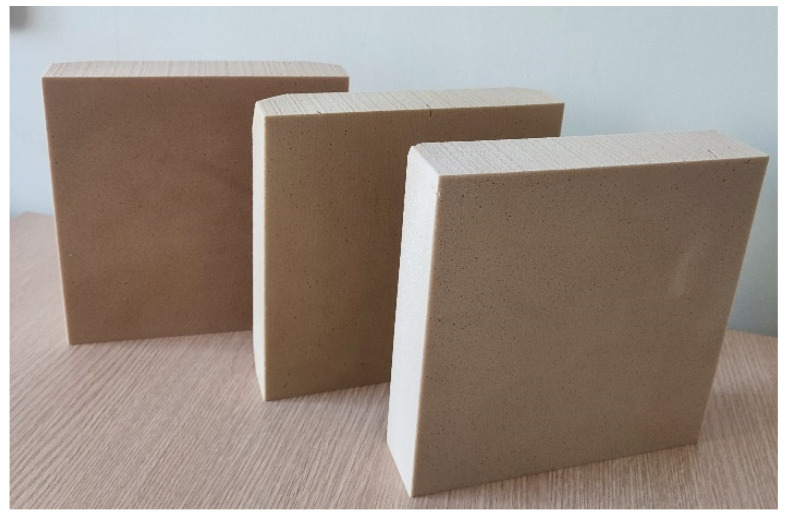
Rigid PU foam samples (20 × 20 × 5 cm). From front to back: SA1-PU, SA2-PU, and SA3-PU.

By visual inspection, the foams exhibited acceptable quality, with a uniform cellular structure free of cracks or other major defects. They were mechanically robust, maintained their shape without deformation, and showed no signs of excessive brittleness. Representative photographs of the samples are shown in Figure 7.

**Figure 8:**
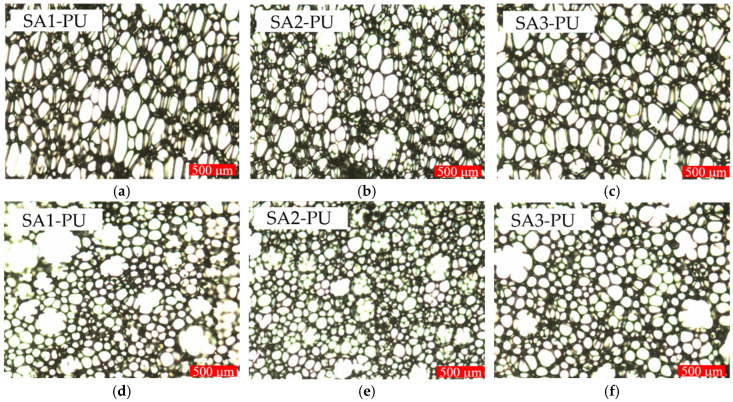
Optical microscopy images showing the cellular morphology of rigid PU foams: (**a**–**c**)—images taken parallel to the foaming direction; (**d**–**f**) images taken perpendicular to the foaming direction.

The cellular structure was further examined by optical microscopy (Figure 8) performed in a parallel direction (a–c). All foams exhibit elongated and anisotropic cells aligned along the foaming direction, which is characteristic of rigid PU foams produced by free-rise expansion. Among the samples, SA2-PU displays the smallest and most uniform cells, whereas SA3-PU contains larger and more elongated cells with a broader cell size distribution.

Foaming parameters, shrinkage, closed-cell content, apparent density, and thermal conductivity are reported in Table 7.

Closed-cell PU foams (closed-cell content > 93%) with apparent densities of 39–45 kg/m^3^ were obtained, corresponding to the typical range of rigid PU foams used in thermal insulation of building materials [22]. Foaming parameters, including start, gel, and rise times, were within optimal ranges, ensuring proper foam formation. SA3–PU system exhibited higher reactivity due to the lower average molecular weight and viscosity of its polyols, resulting in foams with lower apparent density. Consequently, less blowing agent would be required to achieve the same apparent density compared with the SA1–PU and SA2–PU systems. Dimensional shrinkage after 24 h was below 6%, indicating negligible deformation. The rigid PU foams contained approximately 31–34% renewable content, including ~16% SA, and if bio-based TMP were used, the total renewable material content could reach 46%.

**Figure 9:**
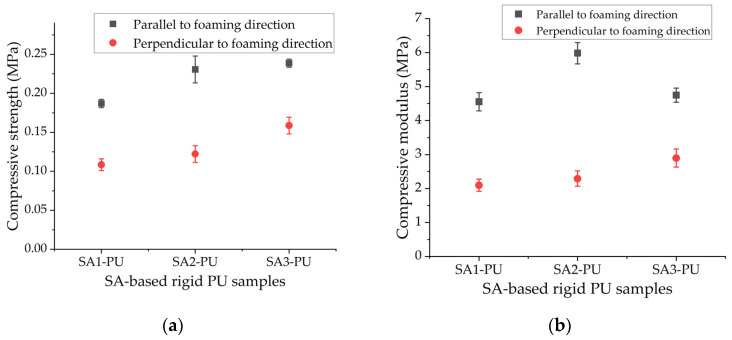
Normalized mechanical properties of SA-based rigid PU foams: (**a**) Compressive strength; (**b**) compressive modulus.

The compressive properties of the SA-based rigid PU foams were evaluated parallel and perpendicular to the foaming direction (Figure 9). All samples exhibited pronounced anisotropy, with compressive strength and modulus measured parallel to the foaming direction being approximately 2–3 times higher perpendicular values, which is typical for closed-cell free-rise rigid PU foams due to preferential cell elongation along the rise direction. SA2-PU and SA3-PU showed comparable compressive strength within the experimental error, whereas SA1-PU exhibited slightly lower values. SA2-PU demonstrated the highest compressive modulus parallel to the foaming direction, indicating a stiffer elastic response. Although SA3-PU had the highest compressive strength, its compressive modulus was lower than that of SA2-PU, likely due to the chemical composition of the SA3 fraction and the resulting polymer network characteristics. The lower viscosity and modified functionality of the SA3-based polyols may have produced a less rigid but more homogeneous crosslinked structure, providing enhanced load-bearing capacity with lower stiffness in the elastic region. Overall, the compressive strength values (0.19–0.24 MPa) are consistent with literature data reported for rigid PU foams with similar apparent densities (40–50 kg/m^3^), including bio-based polyol systems such as SA-based polyols (0.20–0.36 MPa) [11,42], tall oil-based polyols (0.21–0.28 MPa) [44], and polyols derived from lignocellulosic biomass (0.22–0.42 MPa) [45].

**Figure 10:**
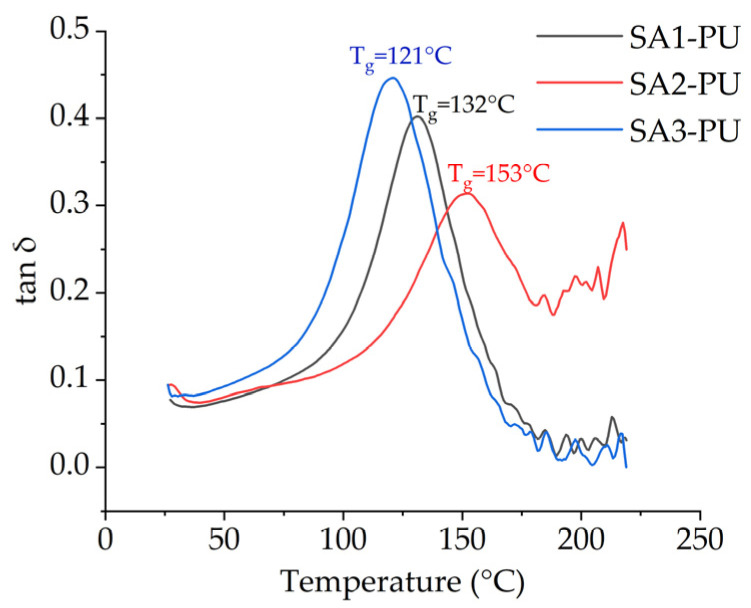
DMA tan δ curves with indicated T_g_.

DMA results for the rigid PU foam samples are shown in Figure 10, with tan δ displayed as a function of temperature.

The T_g_ of each sample was determined from the peak maximum of the tan δ curve. SA3-PU exhibited the lowest T_g_ (121 °C), followed by SA1-PU (132 °C), while SA2-PU showed the highest T_g_ (153 °C). These differences reflect variations in polymer chain mobility: the higher T_g_ of SA2-PU indicates more restricted segmental motion, likely due to increased crosslinking density or a stiffer molecular architecture, whereas the lower T_g_ of SA3-PU suggests a more flexible polymer network. During FeCl_3_ treatment, removal of polyphenolics in SA3 may co-precipitate suberin oligomers rich in hydroxyl or carboxyl groups, reducing the overall functionality of the remaining SA fraction and lowering crosslink density in the resulting PU foam. Although polyphenolics are not the dominant factor controlling T_g_, their residual presence in SA1 and SA2 may still contribute to rigidity through side reactions with isocyanates. In addition, FeCl_3_ treatment may enrich SA3 in shorter aliphatic monomers that act as internal plasticizers, further increasing chain mobility and reducing T_g_.

**Figure 11:**
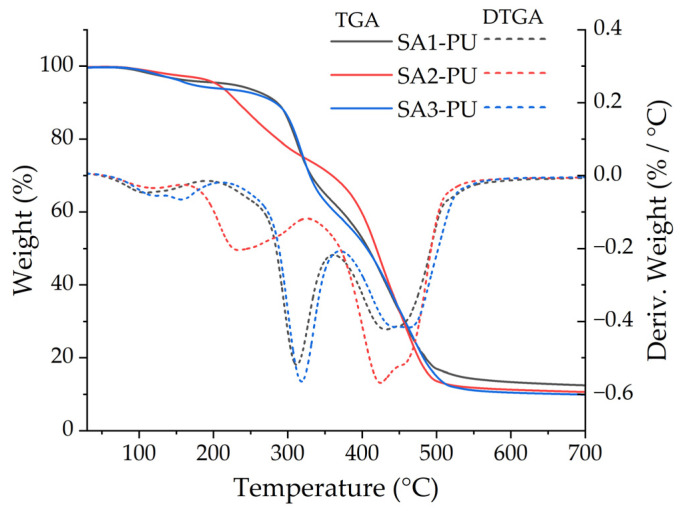
TG and DTGA curves of SA-based rigid PU foams.

To assess the potential effect of polyphenolic compounds present in SA-based polyols on the flammability and thermal stability of rigid PU foams, a series of tests were conducted, including TGA, small-flame ignition tests, LOI, and cone calorimetry. TGA and its derivative DTGA were used to evaluate the thermal stability of the PU foam samples (see Figure 11 and Table 8).

All three samples exhibit a multi-step thermal degradation typical of PUs. The initial weight loss between 80 °C and 200 °C is attributed to the removal of adsorbed moisture, residual low-molecular-weight volatiles, and small amounts of unreacted monomeric species (e.g., foaming agent), as commonly observed in PU systems [46]. SA3-PU exhibits a higher mass loss in the first degradation stage, reaching 5% weight loss at an earlier temperature than other PU samples. The second major mass-loss event occurs between 200 and 400 °C for SA1-PU and SA3-PU, displaying prominent DTGA peaks, corresponding to cleavage of the more labile ester side-chains from the TOFA component and initial dissociation of urethane bonds (allophanate, biuret, and urethane linkages) [46,47,48]. Peak maxima in this region were observed at ~320 °C for SA1-PU and ~340 °C for SA3-PU, indicating relatively high thermal stability. In contrast, SA2-PU exhibits significantly lower thermal stability in this region. SA2-PU showed an earlier onset of the second degradation stage (~120 °C), with a DTGA peak at lower temperature (~235 °C).

The third degradation stage, associated with decomposition of char residue and remaining polymeric fragments (e.g., flexible polyol segments), occurs above 400 °C [49,50].

**Figure 12:**
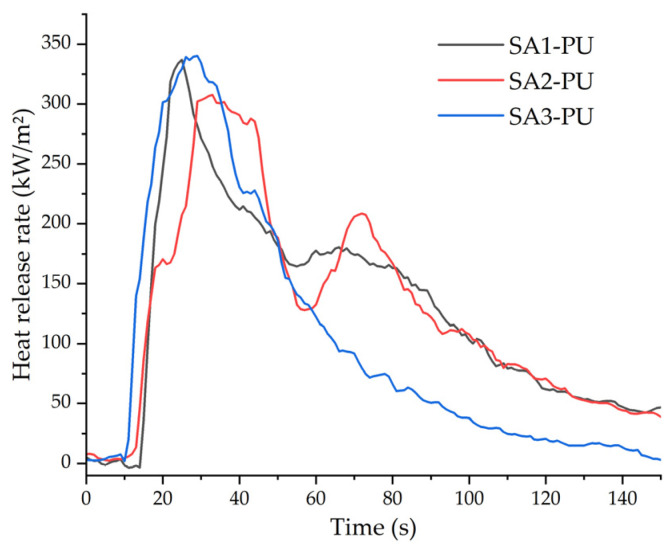
HRR of SA-based rigid PU foams measured by cone calorimetry.

The flammability of the SA-based rigid PU samples was evaluated using the small-flame test (flame height up to 150 mm and afterflame time), the LOI test, and the cone calorimeter test, including TTI, TTF, THR, pHRR, TSR, and MARHE. The corresponding results are summarized in Table 9 and Table 10 and Figure 12.

In the small-flame test, all samples reached a flame height of 150 mm, placing them in the same reaction-to-flame class (F). As a result, this test provided limited ability to distinguish between the materials. Among the samples, the SA2–PU sample showed the longest afterflame time (194 s), indicating a greater tendency for sustained burning after ignition compared to SA1–PU (103 s) and SA3–PU (100 s).

The LOI values of the samples were similar, ranging from 19.1 to 20.1. This places all SA-based rigid PU foams in the flammable category, as materials with an LOI value below 21% are considered flammable, those with between 21% and 2% are combustible, and those above 28% are flame-retardant [51]. These results are consistent with typical LOI values reported for unmodified rigid PU foams without flame retardants, which generally fall within 16–20% [52,53].

In the cone calorimeter test, the pHRR were similar across all samples. SA1–PU and SA2–PU showed somewhat higher THR and TSR values, likely due to warping of the specimens during burning, which increased the effective surface area exposed to the heat flux. This deformation is visible as a secondary peak in the HRR curves; therefore, pHRR provides a more reliable measure for comparison. Based on pHRR, the flammability behaviour of the foams did not depend on the specific suberin extraction method used prior to polyol synthesis, as all samples exhibited values between 323 and 347 kW/m^2^, which lies within experimental variability. The TSR and MARHE values were similarly comparable, ranging from 579 to 727 m^2^/m^2^ and 175 to 211 kW/m^2^, respectively.

Overall, no statistically significant differences in flammability were observed among the tested samples, indicating that variations in suberin depolymerization conditions, including changes in polyphenolic content and the removal of higher-molecular-weight fractions during FeCl_3_ treatment, do not play a dominant role in governing fire performance under the studied conditions. These findings suggest that the concentration and chemical nature of polyphenolic compounds present in the investigated SA fractions are insufficient to induce measurable flame-retardant effects in rigid PU foams. This may be attributed to the relatively low absolute content of polyphenolic structures in the investigated SA fractions and their limited ability to alter the dominant thermal degradation pathway of the PU matrix. Consequently, the combustion behaviour of the foams is primarily governed by the PU network itself, masking any minor contribution arising from differences in polyphenolic content. Additional flame-retardant strategies or higher aromatic/phenolic loadings would therefore be required to achieve meaningful improvements in fire resistance in SA-based PU systems.

## 4. Conclusions

This study demonstrates that suberinic acids derived from birch outer bark, an abundant forestry by-product, can be effectively valorised into bio-based polyols suitable for rigid PU foam production, achieving renewable material contents of up to 46%. The results confirm that depolymerization conditions play a key role in controlling the molecular weight distribution and hydroxyl group content of suberinic acids, thereby governing polyol processability and foam performance.

FeCl_3_-assisted treatment produced a fraction enriched in low-molecular-weight compounds, yielding lower-viscosity polyols with improved processing characteristics while maintaining comparable mechanical and thermal properties. In contrast, SA1-derived systems offer higher yield and stable performance, representing a more resource-efficient alternative.

Importantly, this study reveals that the polyphenolic content naturally present in suberin-derived fractions does not significantly influence flammability within the investigated range, indicating that the intrinsic flame-retardant effects of polyphenolic components are limited under these conditions. This behaviour may be attributed to the relatively low concentration of polyphenolic structures and their limited influence on the dominant degradation behaviour of the PU network. Therefore, higher aromatic/phenolic loadings or additional flame-retardant strategies would likely be required to achieve meaningful improvements in fire resistance.

Overall, the findings highlight the potential of bark-derived suberinic acids as sustainable feedstocks for PU materials and support their role in advancing circular bioeconomy approaches by converting low-value biomass into high-performance polymer systems. Future work should focus on incorporating targeted flame-retardant strategies to enhance material performance further.

## Figures and Tables

**Table 1:** Composition of rigid PU foam formulations.

Component	SA1-PU	SA2-PU	SA3-PU
SA1_TOFA	67.0	-	-
SA1_ETOFA	33.0	-	-
SA2_TOFA	-	67.0	-
SA2_ETOFA	-	33.0	-
SA3_TOFA	-	-	67.0
SA3_ETOFA	-	-	33.0
Total water	0.5	0.5	0.5
PC CAT TKA 30	0.5	0.5	0.5
Polycat NP10	1.55	1.55	1.55
L-6915	2.5	2.5	2.5
Opteon 1100	24.3	24.3	24.3
pMDI	88.4	102.7	101.4

**Table 2:** SA sample characterization.

Sample	Dry Mass Content, %	Yield, %	H_v_, mg KOH/g	Total Polyphenolic Compounds Content, %
SA1	32.5 ± 0.7	50.5 ± 1.7	90 ± 3	3.84 ± 0.19
SA2	46.4 ± 0.9	46.2 ± 1.2	90 ± 3	2.71 ± 0.14
SA3	74.2 ± 1.6	22.8 ± 0.7	106 ± 3	1.12 ± 0.05

**Table 3:** Identification and relative area percentage of monomers in SA samples detected by GC-MS.

Peak	Compound	Relative Area %		
		SA1	SA2	SA3
1	Octadecanoic acid, 9,10—epoxy-18-methyl ester	-	-	0.8
2	Stearic acid	-	-	1.03
3	Ethyl stearate	4.64	3.45	7.15
4	11,14-Eicosadienoic acid	-	-	2.47
5	Hexadecanoic acid methyl ester	13.21	13.22	29.34
6	cis-13-Eicosenoic acid	-	4.65	-
7	Lupeol	-	-	5.65
8	Betulin	82.15	78.68	53.56

**Table 4:** GPC-RID molar mass range and relative area percentage data for SA samples.

Sample	Relative Area, %					
	Molar Mass Range, Da					
	200–500	500–800	1000–2500	2500–3000	3000–5000	>10,000
SA1	24	9	29	-	18	20
SA2	36	8	22	-	16	18
SA3	42	30	24	3	1	-

**Table 5:** SA-based polyols characterization.

Polyol	H_v_, mg KOH/g	OH_v_, mg KOH/g	Water Content, %	Apparent Viscosity, mPa·s	SA Content, %	Renewable Components, %
SA1_TOFA	7.3 ± 1.4	248 ± 3	0.020 ± 0.001	6280 ± 120	35.8	71.7
SA1_ETOFA	3.6 ± 0.5	341 ± 4	0.047 ± 0.002	461,000 ± 13,000	37.3	74.6
SA2_TOFA	8.7 ± 2.3	291 ± 3	0.020 ± 0.003	9250 ± 130	35.8	71.7
SA2_ETOFA	5.9 ± 0.6	406 ± 5	0.010 ± 0.005	286,000 ± 12,000	37.3	74.6
SA3_TOFA	6.9 ± 1.6	291 ± 3	0.012 ± 0.002	3090 ± 80	35.4	70.7
SA3_ETOFA	5.8 ± 0.9	392 ± 3	0.022 ± 0.001	144,000 ± 12,000	36.8	73.6

**Table 6:** GPC-RID (molar mass range and relative area percentage) data for SA-based polyol samples.

Polyol	Relative Area Percentage, %					
	Molar Mass Range, Da					
	200–500	500–800	1000–2500	2500–3000	3000–5000	>10,000
SA1_TOFA	7	9	32	-	23	21
SA1_ETOFA	7	10	15	18	20	24
SA2_TOFA	7	10	40	21	-	25
SA2_ETOFA	7	11	27	-	31	17
SA3_TOFA	10	11	46	-	26	-
SA3_ETOFA	8	11	27	15	15	16

**Table 7:** Selected properties of SA-based rigid PU foams.

	SA1-PU	SA2-PU	SA3-PU
SA content, %	16.7	15.7	15.5
Total renewable material content, %	33.6	31.5	31.3
Foaming start time, s	45 ± 5	36 ± 9	26 ± 1
Foam gel time, s	135 ± 8	64 ± 7	110 ± 8
Foam rise time, s	188 ± 10	89 ± 5	119 ± 5
Shrinkage, %	5.6 ± 0.3	1.1 ± 0.4	1.2 ± 1
Closed cell content, %	93.0 ± 1.7	93.6 ± 0.6	95.6 ± 0.7
Apparent density, kg/m^3^	45 ± 1	42 ± 1	39 ± 1
Thermal conductivity, mW/(m·K)	20.6 ± 0.3	19.9 ± 0.4	20.5 ± 0.2

**Table 8:** SA-based rigid PU foams’ thermal stability characterization.

	SA1-PU	SA2-PU	SA3-PU
First Onset, °C	80 ± 5	92 ± 6	95 ± 6
Temperature at a weight loss of 5%, °C	231 ± 7	202 ± 3	164 ± 17
Temperature at a weight loss of 10%, °C	284 ± 5	232 ± 1	277 ± 13
Temperature at a weight loss of 25%, °C	320 ± 2	318 ± 3	322 ± 2
Temperature at a weight loss of 50%, °C	408 ± 3	419 ± 1	407 ± 3
Solid residue at 700 °C, %	13 ± 1	11 ± 1	11 ± 1
T_DTGA peak1_, °C	104 ± 2	121 ± 6	157 ± 2
T_DTGA peak2_, °C	313 ± 1	234 ± 2	320 ± 1
T_DTGA peak3_, °C	435 ± 4	425 ± 1	465 ± 3

**Table 9:** The flammability properties of the SA-based rigid PU samples determined by small-flame ignition and LOI tests.

Sample	Apparent Density, kg/m^3^	Afterflame Time, s	LOI
SA1-PU	43.5 ± 0.4	103 ± 4	19.7 ± 0.4
SA2-PU	40.5 ± 0.2	194 ± 10	20.1 ± 0.6
SA3-PU	37.7 ± 0.7	100 ± 8	19.1 ± 0.5

**Table 10:** The flammability properties of SA-based rigid PU samples determined by cone calorimeter.

Sample	TTI, s	TTF, s	THR, MJ/m^2^	pHRR, kW/m^2^	TSR, m^2^/m^2^	MARHE, kW/m^2^
SA1-PU	6 ± 3	235 ± 30	21.2 ± 1.1	329 ± 36	727 ± 12	177 ± 20
SA2-PU	7 ± 5	201 ± 33	21.59 ± 0.33	323 ± 62	790 ± 52	175 ± 6
SA3-PU	3 ± 1	166 ± 49	16.0 ± 2.0	347 ± 14	579 ± 33	211 ± 8

## Data Availability

The original contributions presented in this study are included in the article. Further inquiries can be directed to the corresponding author.
